# DRAVP: A Comprehensive Database of Antiviral Peptides and Proteins

**DOI:** 10.3390/v15040820

**Published:** 2023-03-23

**Authors:** Yanchao Liu, Youzhuo Zhu, Xin Sun, Tianyue Ma, Xingzhen Lao, Heng Zheng

**Affiliations:** School of Life Science and Technology, China Pharmaceutical University, 24 Tongjiaxiang, Nanjing 210009, China

**Keywords:** virus, antiviral peptides, database, webserver

## Abstract

Viruses with rapid replication and easy mutation can become resistant to antiviral drug treatment. With novel viral infections emerging, such as the recent COVID-19 pandemic, novel antiviral therapies are urgently needed. Antiviral proteins, such as interferon, have been used for treating chronic hepatitis C infections for decades. Natural-origin antimicrobial peptides, such as defensins, have also been identified as possessing antiviral activities, including direct antiviral effects and the ability to induce indirect immune responses to viruses. To promote the development of antiviral drugs, we constructed a data repository of antiviral peptides and proteins (DRAVP). The database provides general information, antiviral activity, structure information, physicochemical information, and literature information for peptides and proteins. Because most of the proteins and peptides lack experimentally determined structures, AlphaFold was used to predict each antiviral peptide’s structure. A free website for users (http://dravp.cpu-bioinfor.org/, accessed on 30 August 2022) was constructed to facilitate data retrieval and sequence analysis. Additionally, all the data can be accessed from the web interface. The DRAVP database aims to be a useful resource for developing antiviral drugs.

## 1. Introduction

Viruses with rapid replication and easy mutation can become resistant to antiviral drug treatment. In the past two decades, there have been various epidemics, including severe acute respiratory syndrome coronavirus (SARS), Middle East respiratory syndrome coronavirus (MERS), Ebola virus, Zika virus, etc. [1,2,3,4]. Additionally, there was an outbreak of the coronavirus disease 2019 (COVID-19), caused by severe acute respiratory syndrome coronavirus 2 (SARS-CoV-2), which spread globally [5]. The pandemic became a great threat to human health. Although scientific advances have successfully discovered vaccines and antiviral drugs for several kinds of viral pathogens, the emergence and re-emergence of mutant epidemics require persistent efforts to discover novel antiviral agents.

Antiviral drugs can be divided into virus-targeting antivirals and host-targeting antivirals [6]. Virus-targeting antivirals mainly play an inhibitory role in a virus’s life cycle, such as inhibiting virus replication and transcription, blocking virus adhesion to host cells, inhibiting the activity of viral enzymes, etc. [7,8,9]. Host-targeting antivirals focus on regulating the function of host factors or other cellular processes in host cells.

The cytokines involved in the innate immune response to virus invasion act as interferons (IFNs), and interleukins have been explored as modulators of viral infections for decades [10,11]. Among them, IFNs were first discovered by Isaacs in 1957 and have been in clinical use since the 1980s [12,13]. The advances in recombinant DNA technology enabled cytokine production and the detection of their protective effect against microbial pathogens in vitro and in vivo, including IL-1, IL-6, TNF, CSF, etc. [14]. Proteins bound to viral particles have also been considered to inhibit virus invasion. For example, mannose-binding lectin (MBL) has previously been shown to bind to the SARS-CoV spike protein, which is complementarily activated by the lectin pathway [15].

Peptides that possess the potential to inhibit viruses are considered antiviral peptides (AVPs) [8]. Compared with traditional small molecule drugs, AVPs are highly specific and effective, with fewer side effects and host toxicity and show great potential as antiviral drugs [16,17,18,19]. Numerous studies have indicated that AVPs exhibit excellent antiviral activities. For example, EKL1C was shown to have broad-spectrum anticoronavirus activity and potent protection of human angiotensin-converting enzyme 2 (hACE2) in transgenic mice against authentic coronavirus infection [20,21]. Currently, antiviral therapies based on peptides and peptide-related antivirals have been confirmed for human immunodeficiency virus (HIV) and hepatitis C virus (HCV). Enfuvirtide (T-20) was the first FDA-approved fusion inhibitor to treat HIV infection, and in a recent study, Ahmadi et al. recommended that enfuvirtide has the potential to enter the clinical trial phase for treating COVID-19 [22,23]. Additionally, boceprevir and telaprevir are synthetic peptides used for treating chronic hepatitis C [24,25]. Meanwhile, several peptidomimetics, peptide-like small molecules, and amino-acid-like derivatives are currently under investigation in clinical trials for treating various viral infections [26]. Brilacidin is a mimetic of defensin being tested under phase II clinical trials for treating COVID-19 (NCT04784897) [27].

Antiviral peptides can be obtained from computational, natural, and biological sources. Among them, the computational approach is an efficient and convenient method of identifying antiviral peptides. Through molecular docking and simulating peptide structures, peptides and peptidemimics with high affinities interacting with the target can be developed and designed, which is more conducive to developing clinical antiviral drugs. In addition, deep learning and machine learning methods have been widely used in identifying antiviral peptides, with various prediction models being published, such as DeepAVP [28] and AVPIden [29]. To facilitate drug development and to identify antiviral peptides, establishing a comprehensive database of antiviral peptides is necessary. In recent years, antimicrobial peptide (AMP) databases such as DBAASP [30], DRAMP [31], and APD [32] have been built and have collected a large number of AMPs with diverse biological activities, such as antiviral, anticancer, antifungal, and so on. These databases greatly contribute to designing and identifying AMPs. However, most of the common AMP databases did not fully annotate the antiviral peptides. Some special databases, such as HIPdb [33] and ACovPepDB [34], are focused on anti-HIV peptides and anticoronavirus peptides and contain relatively few entries. The special antiviral peptide databases, such as AVPdb [35], have not been updated since 2014. Additionally, antiviral proteins and clinical information have not been included in AVPdb. To promote bioinformatics and assist experimental researchers working in the field of AVPs and antiviral-protein-based therapeutics, a complete data repository of antiviral peptides and proteins was established.

In this study, we developed a comprehensive data repository of antiviral peptides and proteins (DRAVP). The database contains information related to the sequence, antiviral activity, structure, and physicochemical properties of antiviral peptides and proteins. Meanwhile, we also provided information about approved and under-investigation drugs for antiviral therapy based on peptides and proteins. The database integrates search and sequence alignment function to facilitate the data analysis. We believe that DRAVP will be an invaluable resource for identifying and designing antiviral peptides. DRAVP is freely available online at http://dravp.cpu-bioinfor.org/ (accessed on 30 August 2022).

## 2. Materials and Methods

### 2.1. Data Collection and Extraction

To develop a comprehensive database of antiviral peptides and proteins, an extensive search was carried out on published articles and public databases. To collect the antiviral peptides, “((virus[Title/Abstract] OR viral[Title/Abstract]) AND (peptide[Title/Abstract] OR peptides[Title/Abstract])) AND (inhibit*[Title/Abstract] OR block*[Title/Abstract])” were used to search PubMed. This search provided 9559 peer-reviewed articles. From these articles, only antiviral peptides with known amino acid sequences and experimental information were manually extracted. For the antiviral proteins, a text search was carried out in UniProt [36] and PubMed using keywords “antiviral protein*”. After that, the proteins with antiviral effects were screened and registered in DRAVP. Eventually, a total of 1986 antiviral peptides and 139 antiviral proteins, along with detailed information such as the sequence, source, name, modifications, IC_50_, EC_50_, etc., were systematically compiled. DRAVP collects all antiviral activity data as much as possible from articles. Additionally, the physicochemical properties of antiviral peptides were calculated using SciDBMaker [37]. For the structure information, the experimental three-dimensional structures were mainly retrieved from PDB through a sequence blast against the PDB [38] database. If an exact match was obtained, the corresponding PDB ID was assigned to the database. In addition, we also served the modifications and stereochemistry for each sequence on the detailed information page.

To support the clinical research of antiviral therapies, information about antivirals based on peptides and proteins that have been approved or are in the clinical stage was extracted from the literature and drug databases. To facilitate user access to more detailed information about clinical and drug entries, we also provided abundant external links for each entry. Additionally, the clinical trials of each entry were compiled in the database.

### 2.2. Structure Prediction and Evaluation

Antiviral peptides’ structures play an important role in drug development. Due to the experiment’s difficulty, we could only collect a small number of experimentally determined structures from the PDB database. For antiviral peptides and proteins lacking experimental structures in DRAVP, AlphaFold [39] was used to predict the structures. The structure parameters for prediction were set as model: monomer; and multiple sequence alignment information (MSA) database: full_dbs (all gene databases). For each entry, five structures were generated, and the best-ranking structure was selected according to the predicted local distance difference test (pLDDT) score, then displayed on the web interface using Mol* Viewer [40]. To evaluate the structure’s accuracy predicted using AlphaFold, we randomly selected 10 antiviral peptides to benchmark. The structure prediction’s accuracy was judged by comparing the predicted structures with the experimentally determined models and calculating the root-mean-square deviation (RMSD). Additionally, Whatcheck [41] and Procheck [42] were used to evaluate the predicted structures’ quality levels. Whatcheck checks the stereochemical parameters of residues in the model and compares the differences between the submitted peptide structure and the normal structure. Procheck evaluates the stereochemical quality of peptide structures by analyzing whether the Φ/Ψ angle of the amino acid residues is in a reasonable region. Both methods provide “error”, “warning”, or “pass” levels for each evaluation indicator.

### 2.3. Database Construction

**Figure 1:**
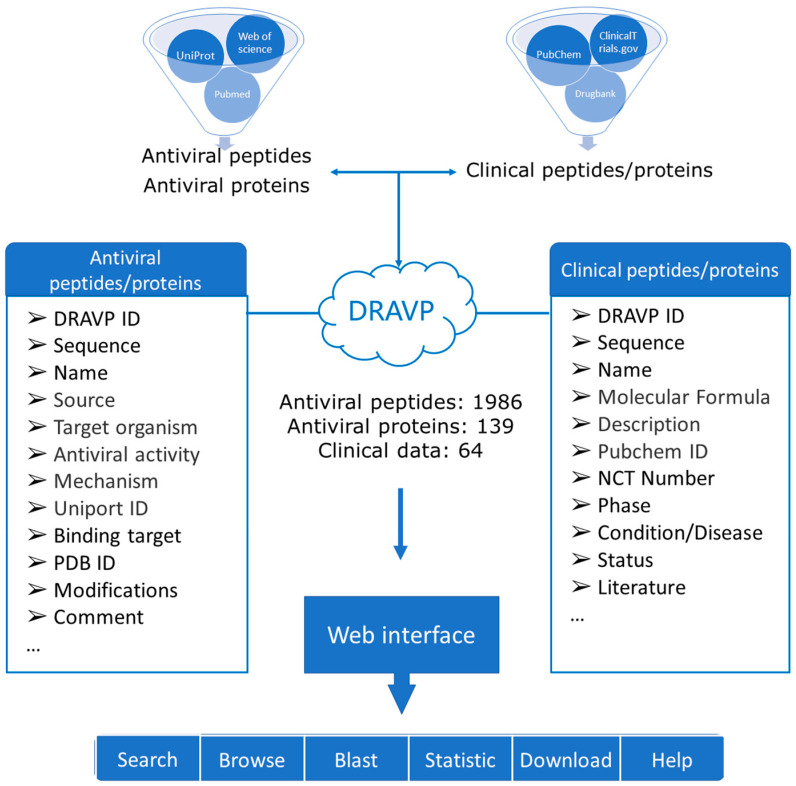
Overview of DRAVP database construction.

The DRAVP web architecture was based on the platform Linux-Apache-MySQL-PHP. After collecting and screening, all the data were stored in the MySQL database as the back end. The visualization software Navicat for MySQL was used to manage the data. Additionally, HTML, PHP, and JavaScript were applied to develop the front-end web interfaces. The database is planned to be updated every three months. To expand the data in the database, a submission page was created for users to submit antiviral peptides and proteins that were not included. We will review the submitted information and add it to our database. The database construction workflow can be overviewed as shown in Figure 1.

## 3. Results and Discussion

### 3.1. Database

**Figure 2:**
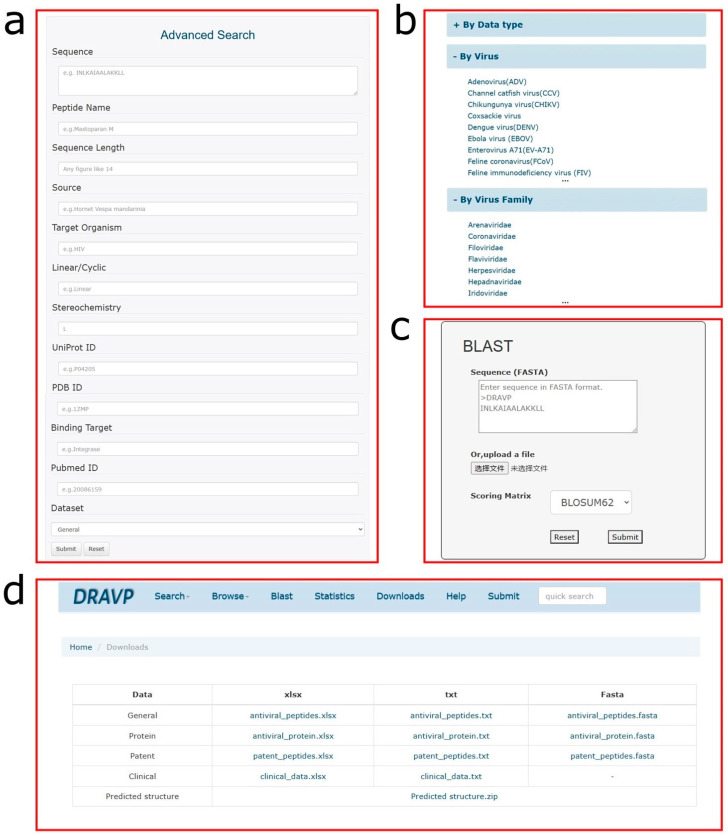
Screenshots of DRAVP web interface. (**a**) The advanced search page, which enables the user to access the desired data through multifield combination retrieval. (**b**) The cross-linked browse page, which provides three classified methods to facilitate browsing: by data type, by target virus, and by virus family. (**c**) The BLAST analysis tool, which enables users to access the most relevant sequence by its local alignments. (**d**) The download page, which provides three types of files of the data and the predicted structures.

DRAVP is freely available at http://dravp.cpu-bioinfor.org/ (accessed on 30 August 2022). Currently, DRAVP contains 1986 antiviral peptides, 139 antiviral proteins, and 64 clinical entries. In the database, 1327 entries of antiviral peptides have predicted structures, while 40 entries have experimentally determined structures from the PDB database. The homepage of DRAVP provides a brief introduction to the database. The navigation bar and quick search tool ensure the use of the website is convenient. The main web page of the DRAVP database contains the following interfaces: Search, Browse, Blast, Statistics, and Download. In addition, users can learn more information from the help page. Figure 2 shows screenshots of the web interfaces, and a brief function description of each page is provided below.

(i) Search page: This page can help users to more conveniently fetch the desired data. To meet different needs, a simple search and advanced search for data retrieval were set up. The simple search allows users to retrieve data from a specific field from the dropdown menu. The advanced search is carried out through a combination of multiple fields, such as sequence, target virus, or UniProt ID, to achieve more accurate data retrieval.
(ii) Browse page: The database provides three cross-linked browse tables based on different criteria: (1) classification based on data type, including antiviral peptides, antiviral proteins, patent data, and clinical data; (2) classification based on targeted virus type, with a total of 47 different virus types; and (3) classification based on virus family, such as coronaviridae, flaviviridae, and retroviridae. Some basic information, such as DRAVP ID, peptide name, source, and target organism, is provided in the overview table.
(iii) Blast: To facilitate the sequence analysis in database, the Blast [43] search tool was integrated into DRAVP. A user can submit sequences in the FASTA format and choose the score matrix to obtain the sequence alignment results, which contain the score, E value, identities, positives, and gaps. The sequence alignment is shown for the peptides or proteins found to be identical or similar in the database.
(iv) Download page: All the data and website source code are freely available for users. Three types of data files are provided on this page to satisfy different requirements. Meanwhile, the predicted structure and installed package of the Blast software are also collected on the page for download.

### 3.2. Database Content

Table 1 shows detailed information related to antiviral peptides and proteins that are displayed on the interfaces. Each entry of the antiviral peptides includes five parts: general information, activity information, structure information, physicochemical information, and literature information. Low cytotoxicity and hemolytics are essential conditions for developing antiviral peptides as drugs. For the convenience of researchers to design antiviral peptides with high activity and low toxicity, we have collected experimental information on cytotoxicity and hemolytic in the database. All the data were manually extracted from articles. Additionally, the data’s exactitude is one of the most important criteria for database evaluation. To prevent any possible errors, we provided literature sources for each included activity data on the web interface. All the data are available on the DRAVP website. In the physicochemical information section, we show each peptide’s amino acid distribution, which is helpful for analyzing the differences and rational design of the antiviral peptides. For antiviral proteins, we also provided comment information, which includes some information such as the half-life and antiviral mechanism.

The clinical data include antiviral drugs based on peptides and proteins that are approved or in the clinical trial stage. Table 2 shows the information displayed on the web interface of the clinical entries. We also provided external links to access various databases, such as PubChem [44], DrugBank [45], and ChEMBL [46], so users can gain more comprehensive information about entries from other sources. The clinical data may serve as data mining examples to help users focus on the most promising drug candidates. At present, the drugs that are approved and under investigation in clinical trials are mainly used for treating human immunodeficiency virus (HIV) infection, hepatitis C virus (HCV) infection, and COVID-19.

### 3.3. Structure Verification

AlphaFold has been verified to be highly accurate in protein structure prediction, but the reliability and accuracy in peptide structure prediction remain to be evaluated. Ten entries with experimentally determined structures were randomly selected from the database to verify AlphaFold’s peptide structure prediction performance. Table 3 shows the results of the Cα RMSDs between the predicted and experimentally determined structures. From the results, we found that the antiviral peptides were predicted with good accuracy and few outliers. The minimum RMSD value was 0.460 Å, and most Cα RMSDs were less than 2 Å. Additionally, the peptides with long sequences had higher accuracies than the short peptides on predicted structures.

According to Whatcheck and Procheck’s results, the predicted structures’ qualities were at high levels (Table 4). The average error rate evaluated by Whatcheck was at a lower level (11.42%). Additionally, in the Ramachandran plot created with Procheck, most residues were distributed in the core regions. Only DRAVPe01958 had one residue (Val18) in the disallowed region, but the Cα RMSD value of the entry between the predicted structure and known structure was 0.460 Å, which explains why the predicted structure is more accurate. McDonald et al. [47] found that AlphaFold predicted α-helical, β-hairpin, and disulfide-rich peptides with high accuracy. Additionally, our research showed that antiviral peptides were mostly α-helical peptides. Therefore, AlphaFold is reliable for peptide structure prediction.

**Figure 3:**
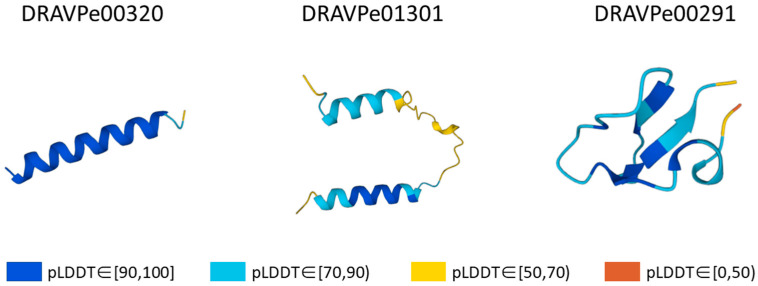
Example outputs containing both high- and low-confidence regions.

Additionally, to know each predicted structure’s exactitude, the Mol* Viewer integrates the detailed information page to display the predicted structure, which allows users to check each amino acid’s pLDDT score. Figure 3 shows some examples containing high- and low-confidence regions. Currently, DRAVP contains 1327 predicted structures and 40 experimentally determined structures from the PDB database.

### 3.4. Statistics

**Figure 4:**
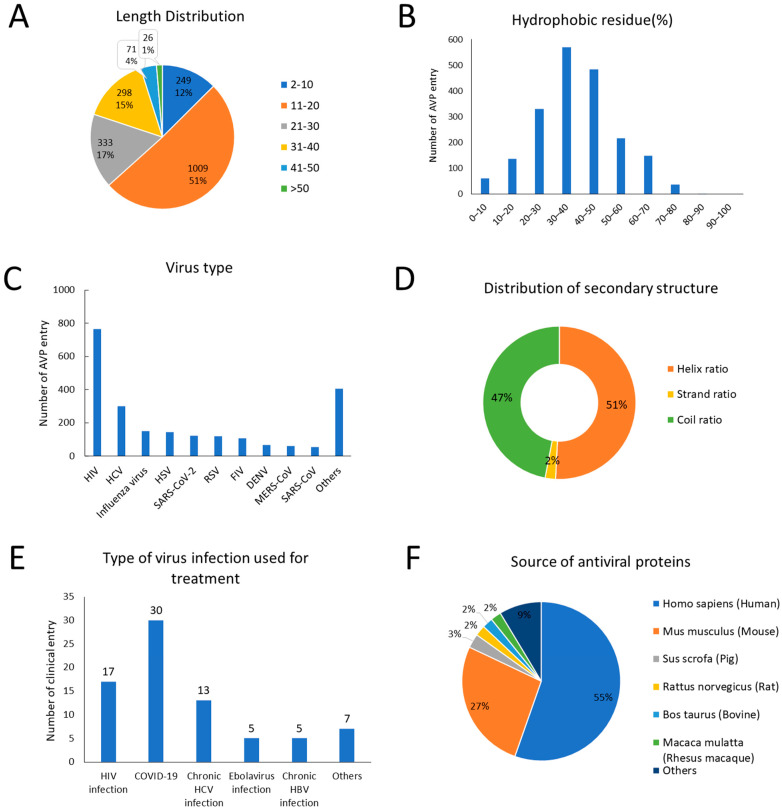
Data statistics in DRAVP database. (**A**) The length distribution of antiviral peptides in DRAVP. (**B**) Hydrophobic content distribution of antiviral peptides. The hydrophobic content represents a ratio between total hydrophobic residues and total amino acids. (**C**) Number of antiviral peptides target various viruses. (**D**) Distribution of secondary structure in predicted structures. (**E**) Type of virus infection that the AVP used for treatment of clinical data. Among them, the number of entries used to treat COVID-19 and HIV infections is largest. (**F**) Source of antiviral proteins.

To further understand antiviral peptides’ features as a basis for peptide design, statistics were calculated in DRAVP (Figure 4). Most peptides consisted of less than 40 amino acid residues. As shown in Figure 4B, the proportion of hydrophobic residues in most peptides was 30–50%, indicating that antiviral peptides in DRAVP are less hydrophobic. By analyzing the results, we found that short peptides with low hydrophobicity were relatively easy to form into antiviral peptides, which may be useful for designing antiviral peptides. Among the antiviral peptides, the number with anti-HIV activity was largest, and there were 124 entries with anti-SARS-CoV-2 activity. According to the secondary structure statistics, the helix is the main component of secondary structures, followed by the coil, and with the strand accounting for the least, which shows that antiviral peptides prefer an α-helix in stable secondary structures. Now the database contains 64 entries of clinical data consisting of 33 proteins, 24 peptides, and other antivirals with peptide structures such as peptidomimetic. As can be seen in Figure 4E, 30 entries are approved or in clinical trials for treating COVID-19, such as Aviptadil, which acts as an interleukin-6 inhibitor for treating COVID-19 (NCT04843761) and is in a phase 3 clinical trial, and Sotrovimab, which is a monoclonal antibody approved for COVID-19 treatment. For antiviral proteins, more than half of the proteins were isolated from humans (55%), with others being extracted from mice, pigs, rats, bovines, and so on. Among these antiviral proteins, IFN and IFN-induced proteins account for the majority, such as IFN-inducible transmembrane (IFITM) proteins and Mx GTPases.

It is a rapid and efficient approach for researchers to design new antiviral peptides with improved activity using the known peptides as templates. Table 5 lists the entries with the highest antiviral activity targeting various viruses in DRAVP, such as SARS-CoV-2, HIV, EBOV, etc. By analyzing antiviral mechanisms, most of the peptides were found to play an antiviral effect by blocking viral entry into host cells, which indicates that preventing viral entry is a promising target for antiviral intervention. These findings may contribute to the rational design of novel peptides with improved activities and the clinical application of antiviral peptides.

## 4. Conclusions

Recently, increasing reports of viral mutation and emergencies require constant work to discover novel antiviral agents. Antiviral peptides exert both preventative and therapeutic functions against viral infection. However, peptide drugs generally have short half-lives and poor stability. To better promote the development of antiviral peptides as drugs, it was necessary to construct a database. Here, we constructed a comprehensive database of antiviral peptides and proteins that aims to be a useful resource for discovering and designing novel antiviral drugs. A user-friendly interface was established to facilitate data searching, browsing, and alignment. The database allows the user to download all the data and source code. DRAVP will continue to collect experimentally determined antiviral peptides and reupdate all existing data. We promise to regularly update the database for at least 5 years.

## Figures and Tables

**Table 1:** List of fields in antiviral peptides dataset.

Column	Fields	Description
General information	DRAVP ID	The unique ID linking to the corresponding DRAVP entry
	Peptide name	Name of each peptide/protein
	Sequence	Amino acid composition of peptide/protein
	Sequence length	The length of peptide/protein sequence
	UniProt ID	The accessing link directing to external UniProt entry
	Source	The organism where the peptide/protein is extracted or isolated
Activity information	Target organism	Type of targeted virus
	Assay	Methods for determining the antiviral activity
	Activity	Antiviral activity information against viruses; contains EC_50_, IC_50_, etc.
	Hemolytic activity	Hemolytic activity against red blood cells
	Cytotoxicity	Cytotoxicity against cells except for RBCs
	Binding target	Peptide active action site
	Mechanism	Mechanism of peptides exhibit antiviral activity
Structure information	PDB ID	Provide accessing link directing to the corresponding PDB entry
	Predicted structure download	Provide the predicted structure file
	Linear/cyclic	Peptide linear or cyclic structure
	N/C-terminal modification	The modifications of N/C-terminal according to the references
	Other modification	All bonds and special amino acids
	Stereochemistry	The L/D amino acids consist of peptides
Physicochemical information	Formula, mass, pI, net charge, absent amino acids, common amino acids, basic residues, acidic residues, hydrophobic residues, Boman index, hydrophobicity, aliphatic index, half-life, extinction coefficient cystines, absorbance 280 nm, and polar residues	
Literature information	Title, PubMed ID, journal information, and doi of reference articles	

**Table 2:** List of fields in clinical dataset.

Field	Description
DRAVP ID	The unique ID linking to the corresponding DRAVP entry
Name	Name of the entry
Sequence	Amino acid composition of peptide
Molecular formula	Molecular formula of the item
Condition/disease	Diseases used for treatment
Group	Clinical trials status
Type	Peptide, protein, or other types of drugs
PubChem ID	The accessing link directing to external PubChem entry
DrugBank accession number	The accessing number in DrugBank database
Description	Some information related to the entry, such as antiviral mechanism, structure description, etc.
Active sequence/structure	The chemical structure or modified sequence
CHEMBL ID	The accessing link directing to CHEMBL database
UNII	The accessing link directing to Global Substance Registration System
CAS	The accessing link directing to CAS common chemistry
Reference	The PubMed ID of reference articles
Clinical trials information	Including NCT number, study title, condition/disease, status, sponsor, and phase of clinical trials

**Table 3:** Comparison between predicted structures and experimental structures.

DRAVP ID	PDB ID	Sequence Length	RMSD(Å)
DRAVPe00279	2ERI	31	0.486
DRAVPe01560	2LZI	18	1.327
DRAVPe00303	1ZMQ	32	0.409
DRAVPe01598	1ULL	17	1.772
DRAVPe00373	2JOS	22	2.158
DRAVPe00837	2MLT	26	1.507
DRAVPe01303	1ZMP	32	0.519
DRAVPe00295	1ZA8	31	0.680
DRAVPe01958	1FD4	41	0.460
DRAVPe00376	6GS5	13	2.305

**Table 4:** The evaluation results of predicted structures using Whatcheck and Procheck.

DRAVP ID	Whatcheck			Procheck		
	Total Metrics	Error	Error Rate	Core Regions	Additional and Generous Allowed Regions	Disallowed Regions
DRAVPe00279	45	5	11.11%	80.0%	20.0%	0.0%
DRAVPe01560	37	6	16.22%	92.9%	7.1%	0.0%
DRAVPe00303	45	5	11.11%	100.0%	0.0%	0.0%
DRAVPe01598	39	5	12.82%	100.0%	0.0%	0.0%
DRAVPe00373	43	4	9.30%	100.0%	0.0%	0.0%
DRAVPe00837	42	4	9.52%	100.0%	0.0%	0.0%
DRAVPe01303	42	5	11.90%	96.3%	3.7%	0.0%
DRAVPe00295	43	5	11.63%	88.9%	11.1%	0.0%
DRAVPe01958	46	5	10.87%	96.7%	0.0%	3.3%
DRAVPe00376	41	4	9.76%	100.0%	0.0%	0.0%
Average			11.42%	95.48%	4.19%	0.33%

**Table 5:** List of antiviral peptides with the highest activity for eight different viruses.

DRAVP_ID	Virus	Target	Mechanism	IC_50_/EC_50_	Reference
DRAVPe00482	SARS-CoV-2	S protein	Prevent viral entry by binding to the spike receptor-binding domain (RBD) and blocking it from binding to ACE2	23.54 pM	Cao et al. [48]
DRAVPe00788	Human immunodeficiency virus (HIV-1 and HIV-2)	Envelope protein	Inhibit viral entry by binding to the prehairpin intermediate (PHI) conformation and competitively prevent its transition to 6-HB	0.43 pM ~0.026 nM	Zhu et al. [49]
DRAVPe01747	Hepatitis C virus (HCV)	Envelope protein	Inhibit viral entry at the postattachment step and block cell-to-cell transmission	1–5 nM	Yin et al. [50]
DRAVPe01288	Dengue virus (DENV1, DENV2, DENV3, DENV4)	-	Interact with the viral membrane bringing peptides into the low-pH endosome, where full exposure of the peptide site on the E conformational intermediate leads to the tight, specific binding that blocks membrane fusion	IC_90_ = 0.1–4 μM	Schmidt et al. [51]
DRAVPe01806	Ebola virus (EBOV)	-	Impair cathepsin B-mediated processing of EBOV glycoprotein, thus preventing virus entry	0.99 µM	Yu et al. [52]
DRAVPe00757	Herpes simplex virus (HSV-1)	-	Inhibit the viral attachment and viral entry of HSV-1	0.67–2.88 µg/mL	Zeng et al. [53]
DRAVPe01977	SARS-CoV	ACE2	Inhibit viral entry by blocking the binding of S protein to ACE2	1.88 nM	Ho et al. [54]
DRAVPe00407	MERS-CoV	S protein	Inhibit viral six-helical bundle (6-HB) formation, thereby preventing viral fusion and entry into host cells	4.2 nM	Xia et al. [55]

## Data Availability

The source code for the DRAVP database website has been uploaded to GitHub: https://github.com/CPUDRAVP/DRAVP (accessed on 15 November 2022).
